# Learning sparse models for a dynamic Bayesian network classifier of protein secondary structure

**DOI:** 10.1186/1471-2105-12-154

**Published:** 2011-05-13

**Authors:** Zafer Aydin, Ajit Singh, Jeff Bilmes, William S Noble

**Affiliations:** 1 Department of Genome Sciences, University of Washington, Seattle, WA 98195, USA; 2 Department of Electrical Engineering, University of Washington, Seattle, WA 98195, USA; 3 Department of Computer Science and Engineering, University of Washington, Seattle, WA 98195, USA

## Abstract

**Background:**

Protein secondary structure prediction provides insight into protein function and is a valuable preliminary step for predicting the 3D structure of a protein. Dynamic Bayesian networks (DBNs) and support vector machines (SVMs) have been shown to provide state-of-the-art performance in secondary structure prediction. As the size of the protein database grows, it becomes feasible to use a richer model in an effort to capture subtle correlations among the amino acids and the predicted labels. In this context, it is beneficial to derive sparse models that discourage over-fitting and provide biological insight.

**Results:**

In this paper, we first show that we are able to obtain accurate secondary structure predictions. Our per-residue accuracy on a well established and difficult benchmark (CB513) is 80.3%, which is comparable to the state-of-the-art evaluated on this dataset. We then introduce an algorithm for sparsifying the parameters of a DBN. Using this algorithm, we can automatically remove up to 70-95% of the parameters of a DBN while maintaining the same level of predictive accuracy on the SD576 set. At 90% sparsity, we are able to compute predictions three times faster than a fully dense model evaluated on the SD576 set. We also demonstrate, using simulated data, that the algorithm is able to recover true sparse structures with high accuracy, and using real data, that the sparse model identifies known correlation structure (local and non-local) related to different classes of secondary structure elements.

**Conclusions:**

We present a secondary structure prediction method that employs dynamic Bayesian networks and support vector machines. We also introduce an algorithm for sparsifying the parameters of the dynamic Bayesian network. The sparsification approach yields a significant speed-up in generating predictions, and we demonstrate that the amino acid correlations identified by the algorithm correspond to several known features of protein secondary structure. Datasets and source code used in this study are available at http://noble.gs.washington.edu/proj/pssp.

## Background

Understanding a protein's functional role often requires knowledge of the protein's tertiary (3D) structure. However, experimentally obtaining an accurate 3D structure can be labor-intensive and expensive, and methods for computationally predicting 3D structure are far from perfect. Therefore, protein *secondary structure *provides a useful intermediate representation between the primary amino acid sequence and the full three-dimensional structure. The secondary structure of a protein is most commonly summarized via a labeling of the amino acids according to a three-letter alphabet: H = helix, E = strand, L = loop. Knowledge of a protein's secondary structure can provide insight into its structural class, suggest boundaries between functional or structural domains, and give clues as to the protein's function. Furthermore, because protein secondary structure prediction is often used as a subroutine in tertiary structure prediction algorithms, any significant improvement in secondary structure prediction is likely to yield improved tertiary structure predictions as well.

The earliest method for secondary structure prediction [1] used a neural network to achieve a base-level predictive accuracy of 64.3% from a dataset of 106 labeled proteins. In the ensuing 22 years, dozens of methods have been proposed for improving upon this baseline, with significant advances achieved by exploiting homologs of the query sequence [2] and by employing methods, such as hidden Markov models, which exploit patterns in the protein sequence [3]. State-of-the-art methods now achieve accuracies in the range of 77-80% on a variety of published benchmark datasets [4].

Our secondary structure prediction method combines a dynamic Bayesian network (DBN) and a support vector machine (SVM). DBNs and SVMs have already been used successfully to predict protein secondary structure [5,6]. A DBN is a type of graphical model, which is an intuitive, visual representation of a factorization of the joint probability distribution of a set of random variables. DBNs are Bayesian networks that can be extended in one dimension to arbitrary lengths. This type of model is therefore ideally suited to handling variable length data such as protein sequences. Indeed, the hidden Markov model, which has been used extensively to model protein sequences [7-11], is a very simple example of a DBN. Generative models such as DBNs and HMMs are also used in modeling torsion angles and in predicting the three-dimensional structure of proteins [12-14]. An SVM is a non-parametric statistical method for discriminating between two classes of data [15,16]. SVMs have been applied widely in bioinformatics [17]. The SVM operates by projecting the data into a vector space and finding a hyperplane that separates the classes in that space. SVMs are motivated by statistical learning theory, which suggests an optimal method for identifying this separating hyperplane. SVMs are thus functionally similar to neural networks, but can be mathematically represented as a convex optimization problem, meaning that the cost function has a single minimum, making it possible to identify a globally optimal solution in an efficient fashion. In this work, we first extend a previously described DBN [5], combine it with an SVM, and introduce several improvements that yield performance comparable to the state-of-the-art on an established benchmark.

In addition to improving the predictive performance of DBNs, we introduce an algorithm for learning a sparse DBN for protein secondary structure prediction. In this context, *sparse *refers to a model in which a large percentage of the model parameters are zero. Methods for encouraging sparsity have been the subject of much recent work in the statistical machine learning community [18-20] because these methods have the potential to learn the trade-off between over- and under-fitting a given data set. In general, a model with many parameters will tend to overfit the training set and therefore fail to generalize to the test set. Conversely, a model with too few parameters will underfit the training data and hence achieve poor predictive power on both the training data and the test set. Ideally, a sparse learning algorithm will be allowed to fit a large number of parameters but, depending on properties of the training set, will choose to set some percentage of those parameters to zero. The sparse learner thus, analogous to a non-parametric model, balances model complexity against training set size, with the goal of balancing between under- and over-fitting. Other technical benefits of the resulting sparse model include improved robustness to new test data and greater efficiency. In addition to technical advantages, sparse models enable us to discover correlations inherent in protein structure, including local correlations among neighboring amino acids as well as non-local correlations among *β *strands or coiled-coil regions. The algorithm we propose in this paper interleaves iterations of the expectation maximization (EM) algorithm [21] with a simple sparsification operation, which is straightforward and very effective.

## Results and Discussion

### Comparison with the state-of-the-art

In our first experiment, we performed a seven-fold cross-validation on CB513, which is a well-known and difficult benchmark dataset with 513 chains and 84,119 amino acids [22]. The details of the cross-validation procedure is explained in "Model training, parameter optimization and testing for cross-validation" section. To assign the true secondary structure labels, we mapped the eight-state representation of secondary structure labels (the raw format available in DSSP [23]) to three states with the following conversion rule: H, G, I to H; E, B to E and S, T, ' ' to L. For the experiments in this section, we did not apply the model sparsification algorithm introduced in this paper.

The results of the seven-fold cross-validation are summarized in Table 1, including the amino acid level accuracy (called *Q*_3 _[24]), the segment overlap score (SOV) [25], and Matthew's correlation coefficients (MCC) [26]. In this table, a variety of secondary structure prediction methods--SVMpsi [27], JNET [28], YASSSP [29], DBNfinal [5], DBNpred (our method), DBNN [5], PSIPRED [30], SVM_D3 [31], DESTRUCT [32], DISSPred [6], and DSPRED (our method)--are evaluated with respect to the CB513 benchmark. We evaluated the statistical significance of the differences between the accuracies (*i.e.*, *Q*_3 _measure) reported in Table 1 using a one-tailed Z-test, which attempts to determine if one proportion is greater (or lower) than another. When we compare our method to DISSPred [6], a 0.3% difference in accuracy yields a *p*-value of 0.062 from a one-tailed Z-test. This difference is not significant when we set the confidence level to 95% or 99%. When we compare our method to DESTRUCT [32], which is 1.0% less accurate than our method, we get a very small *p*-value (truncated to zero) in a one-tailed Z-test.

**Table 1:** Comparison of secondary structure prediction methods on the CB513 benchmark dataset

Method	Q_3_(%)	SOV(%)	MCC_H_	MCC_E_	MCC_L_
SVMpsi	76.6	73.5	0.68	0.60	0.56
JNET	76.9	N/A	N/A	N/A	N/A
YASSPP	77.8	75.1	0.58	0.64	0.71
DBNfinal	76.3	72.7	0.71	0.61	0.57
DBNpred	77.3	73.0	0.74	0.61	0.59
DBNN	78.1	74.0	0.74	0.64	0.60
PSIPRED	78.2	77.3	N/A	N/A	N/A
SVM_D3	78.4	N/A	N/A	N/A	N/A
DESTRUCT	79.4	77.5	N/A	N/A	N/A
DISSPred	80.0	N/A	0.77	0.68	0.62
DSPRED	80.3	77.7	0.78	0.68	0.63

Therefore, the 1.0% accuracy difference between our method and DESTRUCT is statistically significant. Furthermore, in our experiments with the SD576 benchmark dataset [5] (see below), we have observed that a one-tailed Z-test when applied to variants of our DBN yields *p*-values < 0.006 for differences in *Q*_3 _on the order of 0.5%, which indicates statistical significance. Therefore among the methods that we tested, our method achieves performance comparable to the state-of-the-art in secondary structure prediction.

In the same benchmark, we also analyzed the contribution of the SVM classifier to the predictive accuracy. To analyze this, we implemented DBNpred, which computes predictions by taking the average of the marginal *a posteriori *distributions from the four DBNs as described in "Combining multiple DBNs." The DBNs in DBNpred are trained on the subset of proteins allocated for DBNs (see "Model training, parameter optimization and testing for cross-validation"). The results in Table 1 show that combining the position specific scoring matrix (PSSM) profiles and the four DBNs using an SVM classifier (DSPRED) performs 3% better according to the *Q*_3_(%) measure and 4% better according to the SOV(%) measure than simple averaging of the *a posteriori *distributions from the DBNs (DBNpred). This difference is statistically significant from a one-tailed Z-test (*p *< 10^-10^) and is mainly due to the following factors. First, the SVM classifier uses the PSSM profiles (PSIBLAST and HHMAKE) as well as the *a posteriori *distributions generated by DBNs, whereas the DBNpred only combines the *a posteriori *distributions to reach a final decision. Therefore, the SVM is learning the relationships among the PSSMs and the *a posteriori *distributions jointly. Second, DBNpred takes a simple averaging of the distributions, but the SVM classifier is able to assign more flexible weights to these features.

In our second experiment, we performed seven-fold cross-validation on SD576, which contains 576 chains and 89,384 amino acids [5], and we compared the performance of our method to DBNfinal and the DBNN methods of Yao *et al. *[5]. Table 2 shows that our method outperforms DBNfinal by 3.4% and DBNN by 1.6% according to the *Q*_3_(%) measure. In *SOV *(%), we outperform DBNfinal by 3.6% and DBNN by 2.3%. This result and the 2.2% increase in *Q*_3_(%) evaluated on the CB513 set (see Table 1) are statistically significant as measured by a one-tailed Z-test (*p *< 10^-10^); hence, our method outperforms the DBN methods of Yao *et al. *[5].

**Table 2:** Comparison of our method and the methods in Yao et al. on the SD576 benchmark dataset

Method	Q_3_(%)	SOV(%)	MCC_H_	MCC_E_	MCC_L_
DBNfinal	78.2	76.8	0.74	0.65	0.60
DBNN	80.0	78.1	0.77	0.68	0.63
DSPRED	81.6	80.4	0.79	0.71	0.65

### Sparsifying the model while maintaining accuracy

**Figure 1:**
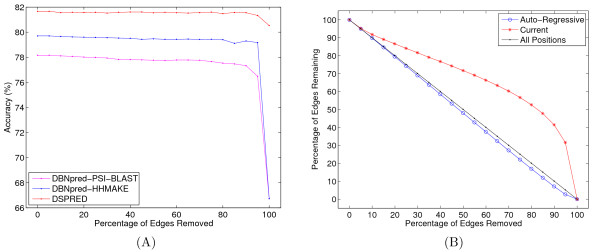
**The effects of sparsification**. (A) The figure plots accuracy as a function of the percentage of dlinks eliminated. Dlinks are the weight parameters that are assigned to the edges in the graphical model representation of the DBN. In an auto-regressive model, the majority of the model parameters become dlink coefficients. It is possible to remove significant proportion of the dlinks while maintaining the overall predictive accuracy such that 70% of the dlink parameters can be removed for the DBN model that uses PSI-BLAST PSSMs only, 80% of the dlink parameters can be removed for the DBN model that uses HHMAKE PSSMs only and the 95% of the dlink parameters can be removed for the DSPRED method (DBN combined with SVM) that uses PSI-BLAST PSSMs, HHMAKE PSSMs and posterior distributions of secondary structure labels. (B) The figure plots the percentage of dlinks that are retained as a function of the sparsity of the model. The three series correspond to all dlinks, only the current dlinks and only the auto-regressive dlinks. In the auto-regressive and the current series, the percentages are computed with respect to the total number of dlinks within each of these series separately. For both panels, results are computed via seven-fold cross-validation on SD576. The hyperparameters of the DBN are *L_AA _*= 5, *L_SS _*= 3, *α *= 0.035, *ω *= 0.4.

A sparse model enables us to control the model complexity and balance between under- and over-fitting against training data. It also brings improved robustness to new test data and greater efficiency. Not all sparsity levels are practically useful mainly because an over-sparsified model will typically have reduced generalization ability and will perform poorly on new test data. Therefore, the primary goal of our study is to develop sparse models that maintain predictive accuracy while reducing the effective number of parameters in the learned model. Accordingly, we measured the extent to which Algorithm 1 (see the "Learning a Sparse Model for a DBN" section) could successfully sparsify a given model. For this experiment, we considered the following three methods: (1) the two DBN classifiers that use PSI-BLAST PSSMs as the input observations (DBNpred-PSI-BLAST) (2) the two DBN classifiers that use HHMAKE PSSMs (DBNpred-HHMAKE), and (3) the DSPRED method with the four DBNs and the SVM. For methods (1) and (2), we performed a seven-fold cross-validation experiment on the SD576 dataset [5], fixing the hyperparameters of the DBNs as *L_AA _*= 5, *L_SS _*= 3, *ω *= 0.4 and *α *= 0.035, where *L_AA _*is the number of positions in the sequence window excluding the current position, *L_SS _*is the length of the secondary structure label window excluding the current label, *ω *is the sequence profile weight, and *α *is the weight of the covariance regularizer (see the "Methods" section for details). For the DSPRED method, we performed a seven-fold cross-validation experiment as described in "Model training, parameter optimization and testing for cross-validation" section, and we used the optimized values for the hyperparameters of the DBN and SVM. Therefore, in this method, the training set allocated for the DBNs is half the training set allocated for the first two methods. For each training set, we first eliminated a specified percentage of the parameters from the DBNs by applying Algorithm 1 and then used the sparse DBN models to compute the *a posteriori *distributions of secondary structure labels. For methods (1) and (2) we computed the final secondary structure prediction by taking the average of the marginal *a posteriori *distributions from the DBNs and for (3) we computed the final prediction by the SVM (see the "Combining multiple DBNs" section). For this experiment, we set *k *= 1%, which corresponds to removing 1% of the edges at the end of each EM iteration, and we considered a range of sparsity values (ℓ = 0, 5, 10, . . . , 100). The results of this experiment are summarized in Figure 1(A), which suggests that we can eliminate 70% of the edge parameters of the DBNs (see the "Graphical model representation" section) when we use PSI-BLAST PSSMs, 80% of the edge parameters when we use HHMAKE PSSMs and 95% of the edge parameters when we use the DSPRED method without significantly decreasing the accuracy of our predictions. To validate this result, we performed a one-tailed Z-test. For method (1), we compared the performance of the fully dense model with the models obtained after removing 70%, 75% and 80% of the edge parameters. Using a significance threshold of 0.01, the performance after removing 70% of the edge parameters--corresponding to a decrease in predictive accuracy of only 0.40%--is not statistically significant (a p-value of 0.021). For 75% removal, the accuracy drops by 0.5% (a p-value of 0.006) and for 80% removal, it drops by 0.63%, (a p-value of 0.001). However, even when removing 80% of the edge parameters, the loss in predictive accuracy is not large. When we remove all the edge parameters (100% sparsity) the accuracy plummets to around 66%. This shows that the sparsification algorithm is removing redundant parameters first, which is a desired behavior for a sparsifier. Once we start removing essential parameters, the accuracy falls quickly. Note that removing all the edge parameters does not correspond to eliminating all the parameters in the DBN model, which explains why the accuracy is not zero (see the "Methods" section). The statistical analysis performed for method (1) can also be performed for methods (2) and (3) but is omitted here for simplicity. When we use HHMAKE PSSMs the performance loss was 0.3% at 80% sparsity and when we use the DSPRED method it was 0.21% at 95% sparsity. Note that the DBN model that uses HHMAKE profiles (method (2)) can generate more accurate predictions (a *Q*_3_(%) of 79.70%) and sparser models than the DBN model that uses PSI-BLAST PSSMs only. Combining both PSSMs by an SVM classifier (the DSPRED method) yields even more accurate predictions (a *Q*_3_(%) of 81.6%) and is more robust to even sparser DBN models as shown in Figure 1(A). Note that even if we eliminate all the edge parameters for the DBNs, the DSPRED method still performs considerably well (a *Q*_3_(%) of 80.50%) because this only causes the *a posteriori *distributions to be less accurate, which is highly compensated by the availability of PSI-BLAST and HHMAKE PSSMs in the SVM's feature set.

### The auto-regressive section of the model contributes to accuracy

The sparsification experiment presented in the previous section also allows us to test the hypothesis that the auto-regressive portion of the model is an important contributor to its accuracy. This hypothesis is most directly supported by the fact that a model with *L_AA _*= 0, *L_SS _*= 0 achieves only 67% accuracy [5]. To investigate more directly the value of the auto-regressive portion of the model, we subdivided the edge parameters into two groups: *current *edges, which connect pairs of amino acids at the current position, and *auto-regressive *edge parameters that connect an element of the PSSM vector at the current position to another PSSM element in a neighboring position. Figure 1(B) plots the percentage of current edge parameters and the percentage of auto-regressive edges that are retained as a function of the sparsity of the DBN model that uses PSI-BLAST PSSMs only (method (1) in the previous section). Not surprisingly, for every sparsity level, the current edges are preferentially retained by the model; on the other hand, even when we eliminate 90% of the edges, the model still contains 7.11% of the auto-regressive edges.

Furthermore, when carrying out this analysis, we observed that, even in extremely sparse models with 80-90% of the edges eliminated, the model still includes edges from all positions within the dependency window. For instance, if the *L_AA _*parameter is chosen as 5, then at 80% sparsity level, edges that remain in the resulting graph stem from all five amino acids that are neighbors of the current amino acid. This observation suggests that even if there is a strong correlation between the current amino acid and those that are three or four residues apart (see the "Local correlations" section), other positions also contain useful correlations that contribute to the predictive accuracy.

### Recovery of true sparse model structures

We have demonstrated that the sparse learning procedure proposed in the "Learning a sparse model for a DBN" section yields a model that provides highly accurate predictions. Next, we would like to verify that the parameters learned by the model are accurate. To address this question, we use simulated data, because the true parameters associated with real data are not known.

Our experiment consists of four steps. First, we learn the parameters of a DBN--state transitions, length distributions and multivariate conditional Gaussians--from real data at different sparsity levels (from 0% up to 20%) using the algorithm described in the "Learning a sparse model for a DBN" section. For this step, we use the SD576 benchmark [5], and we set the model hyperparameters to *L_AA _*= 5, *L_SS _*= 0, *ω *= 1.0 and *α *= 0.0. Second, we use each trained model to generate a series of synthetic data sets of various sizes (100, 250, 500, 1000, 5000, 15000, and 30000 proteins) by sampling from the parameters of DBN. Third, we use the synthetic proteins to learn sparse models, again employing the algorithm in the "Learning a sparse model for a DBN" section. As in step one, we consider a range of sparsity levels (0% to 20%), and we also train from different numbers of synthetic proteins. Finally, in step four, we compare, for each model, the true underlying parameters and the inferred parameters. In this experiment, we only considered the past dependency DBN in the "Combining multiple DBNs" section and we utilized PSSMs derived from PSI-BLAST [33].

**Figure 2:**
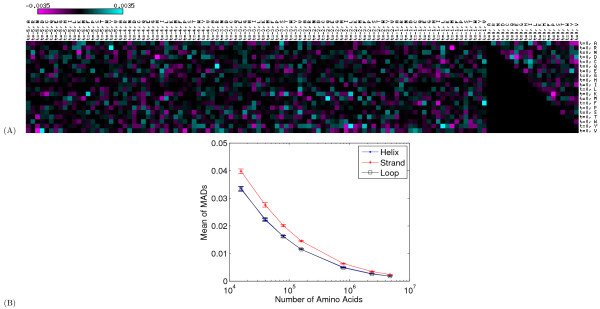
**Comparison of learned and true dlink values**. (A) The figure depicts the difference between the dlink values learned from real data and synthetic data with 30,000 proteins for helices at 20% sparsity level (see Figure 4(A) for a more detailed representation of this result). (B) The figure plots, as a function of training set size, the mean of the MAD metric (see text) computed across ten replicate experiments with 20% sparsity. The three series correspond to the helix, strand, and loop graphs. Error bars correspond to standard deviations. Both figures demonstrate that the model parameters learned from synthetic data are close to the parameters learned from real data. This shows that when the data is generated from a sparse model (*i.e.*, the true model we are trying to recover is sparse) the sparsity algorithm proposed in this paper is able to learn these parameters correctly.

**Figure 3:**
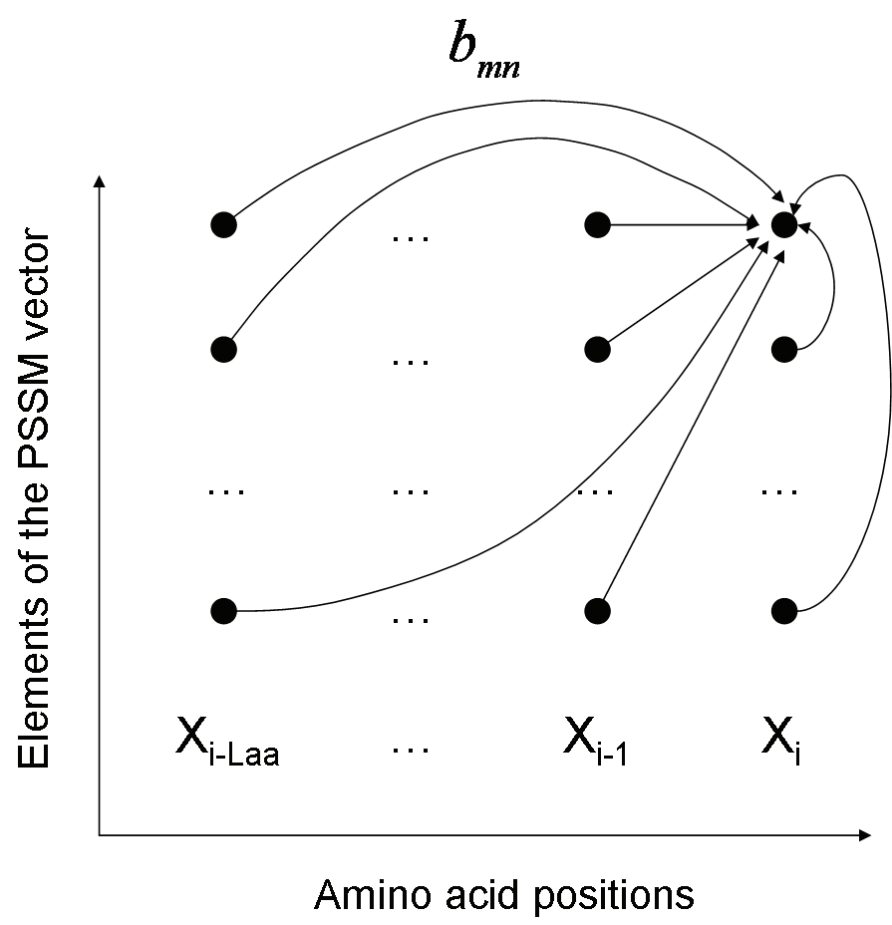
**Schematic of the dlink structure, used to represent correlations in the DBN**. Each node represent a PSSM element and edges represent the dlink coefficients of the DBN. The rightmost column corresponds to the *i^th ^*vector of the PSSM X_i _(*i.e.*, the vector for the *i^th ^*position along the amino acid sequence). The remaining columns represent the columns of the PSSM that come before the *i^th ^*position if we traverse the sequence from the N-terminal to the C-terminal of the protein. Rows represent the 20 amino acids in the PSSM. For simplicity only edges for the child node *x_i_*(1) is shown, which is the first element of the observation vector at the *i^th ^*position. The child nodes are in the rightmost column and the parent nodes are in positions *i - L_AA_*, ..., *i*.

Figure 2(A) shows the difference betweeen the true and learned parameters for one particular DBN. These values are associated with the edges of a graph, as illustrated in Figure 3. Because we set *L_SS _*= 0, we have a total of three such graphs to learn, one for each secondary structure type. The matrix in Figure 2(A) corresponds to the helix graph structure learned from 30,000 proteins with 20% sparsity. In the figure, rows represent the elements of the PSSM vector at position *i *and columns represent elements of the PSSM vectors at positions *i *- 5 to *i *with column indices increasing from left to right. From this figure, we can see that most of the edge differences are close to zero, implying that the learned parameters are close in value to the true values.

**Figure 4:**
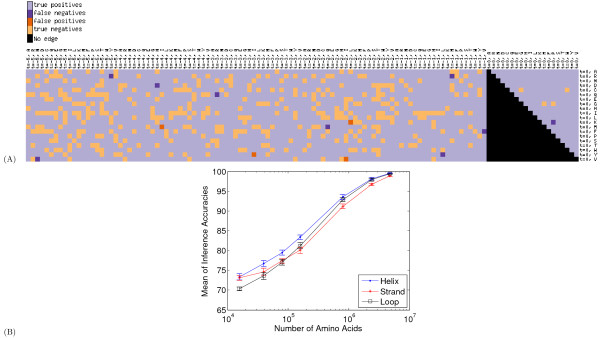
**Comparison of learned dlink structure and true dlink structure**. (A) This is a discretized version of Figure 2(A). For a given pair of matrices, we can characterize each edge as a *true positive *if it occurs in both the true and inferred dlink matrix, a *true negative *if it occurs in neither, or a *false positive *or *false negative *if it occurs only in the inferred or only in the true matrix, respectively. In the resulting sparse model, an edge (*i.e.*, dlink) is counted as occurring when the absolute value of its dlink coefficient is greater than zero. Otherwise, it is counted as non-occurring. In the figure, each dlink is colored according to whether it is a true positive, false positive, true negative or false negative. Overall, this particular inference procedure yields 4 false positives, and 9 false negatives from a total of 2190 possible edges, for an accuracy of 2177/2190 = 99.4%. (B) The figure plots inference accuracy ((TP + FP)/(TP + FP + TN + FN)) as a function of training set size, for a fixed sparsity level of 20%. As the size of the training set grows, the algorithm is able to converge to the true sparse model.

To provide a more quantitative estimate of the difference between the learned and the true edge parameters, we repeated the synthetic data generation experiment ten times. We then averaged the absolute values of the edge parameter differences across all of the parameters in the model, which is called the mean absolute difference (MAD) metric. Figure 2(B) plots the mean and standard deviation of the MAD metric across the ten replicate experiments. The figure shows that, as the sample size increases, the MAD metric decreases. For the largest dataset, the average difference between the true and inferred parameters is very small (0.00183 for helix, 0.00234 for strand and 0.00183 for loop). Figure 4 provides an alternative way of comparing the true and the inferred edge parameter values, based on comparing the inferred graph structures. Both sets of results demonstrate that, given sufficient training data, the sparsification algorithm can successfully infer the correct graph structure.

### Sparse DBNs identify significant correlations among amino acids

One motivation for employing sparse models is the improved interpretability of a model with fewer parameters. Therefore, to complement the simulation experiment described in the previous section, we analyze the graphs of DBNs (past and future dependency models with PSI-BLAST PSSMs) learned from real protein sequences, searching for evidence of various correlations that occur in different types of secondary structure. Therefore, in this section, we are not generating any secondary structure predictions but training a DBN only and sparsifying the graphical model of the PSSM profiles. In this type of analysis, the edges that remain in the sparsified model will represent the particular pairs of PSSM elements that are strongly correlated.

#### Local correlations

It is well known that, in helices, there is a hydrogen bond between every three or four amino acids, depending on the type of helix (excluding the rare type that has bonds every five residues). This bonding pattern causes pairs of helix amino acids that are three and four residues apart to be statistically correlated. Similarly, in *β *strands, amino acid pairs that are adjacent and those that are separated by one amino acid are strongly correlated due to hydrogen bonds and chemical interactions. In contrast, the correlations in loops are more irregular, with the highest correlation occurring between the adjacent amino acids.

**Figure 5:**
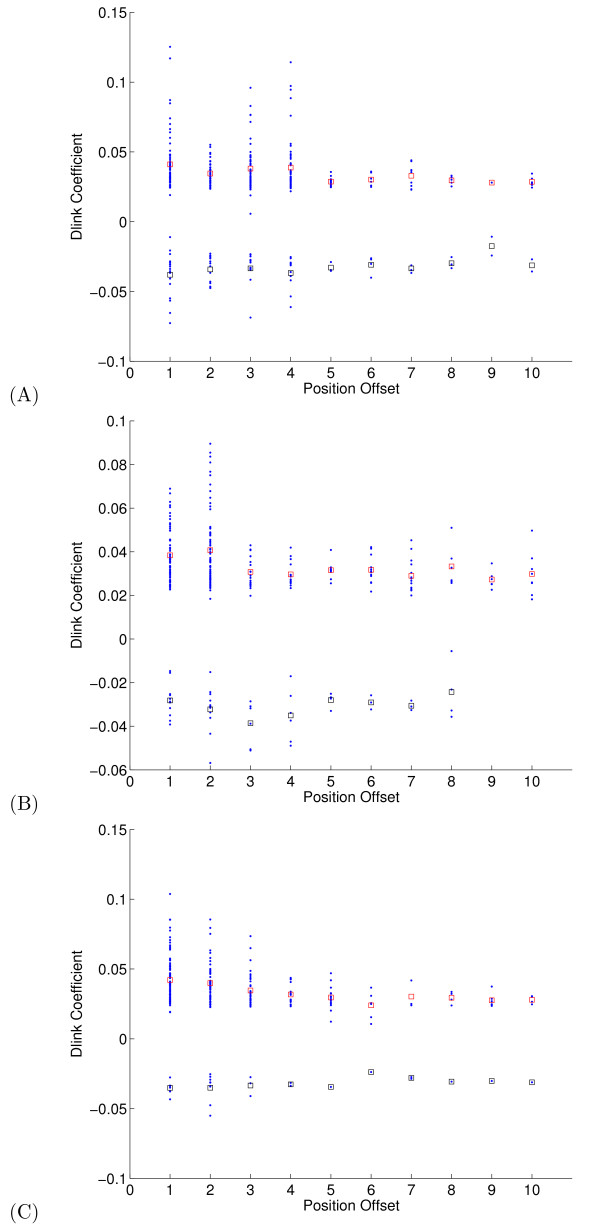
**The relationship between model parameters and secondary structure element types**. (A) The figure plots, for helices, the learned non-zero dlink parameters at the 90% sparsity level as a function of the offset from the current amino acid. The mean of positive (red squares) and negative values (black squares) are displayed separately. (B)-(C) The figures show similar plots for *β *strands and loops, respectively. Certain positions retain more dlink parameters than the others demonstrating the correlation behavior between a given position and its local neighbors.

To assess the relation between the learned graph and these known statistical correlations, we first set *L_SS _*= 0, so that we have one Gaussian for each type of secondary structure element. We chose the input observation window *L_AA _*= 10 so that we cover a wide range of local correlations. Other parameters of the DBN are selected as *ω *= 1.0 and *α *= 0.0 for simplicity. Then we learned the parameters of the model on the SD576 benchmark and sorted the edges with respect to the edge coefficients. The results, summarized in Figure 5, show good agreement with the expected correlation structure. It can be observed that in helices, edges with separation distances of 1, 2, 3, 4 and 7 have high edge coefficients as compared to the other offset values. A similar pattern is obtained for the mean values of the edge coefficients. Furthermore, most of the remaining edge coefficients in the resulting sparse model fall into one of these five offset bins. For *β *strands and loops, edges whose vertices come from adjacent positions as well as positions that are separated by one amino acid had high coefficient values. These results show that the sparse models can be used to capture the biological and statistical correlations that are characteristic of local secondary structure.

#### Non-local correlations in β strands

The chemical interactions in *β *strands differ from those in helices and loops. Specifically, in helices and loops, interactions are primarily local with respect to the amino acid backbone, whereas *β *strand interactions are both local and non-local. The non-local interactions in *β *strands arise mainly due to hydrogen bonds between amino acid pairs positioned in interacting *β *strand segments. We hypothesize that some of the remaining error in our secondary structure predictions--the difference between 80% accuracy and 100% accuracy--results from the failure of our model to capture these non-local interactions. To assess the extent to which such interactions occur and could in principal be captured by our model, we carried out an experiment in which we provided the DBN with additional information about the location of *β *strand interactions. We then measured the extent to which these non-local interactions yield correlation structure in the model. For training, we collected a set of 3,824 protein chains. This dataset, called PDB-PC15, was obtained using the PISCES server [34] (see the "PDB-PC15 dataset" section for details). To analyze the non-local correlations in *β *strands we modified the probability density that is normally used in DBN to model the generation of PSSMs from each secondary structure segment. Details of this updated version of the model can be found in the "Model for analyzing correlations in *β *strands" section. Having designed the model and the dataset, we set *L_SS _*= 0 and applied the sparsity algorithm, eliminating 80% of the edges from the graphical model. Because our dataset contains no helices or loops, the algorithm sparsifies the graph for *β *strands only. In this experiment, we used PSI-BLAST's PSSM profiles only as the observation data (see the "Generating position-specific scoring matrices" section), and set the other hyperparameters to *L_AA _*= 5, *ω *= 1.0 and *α *= 0.0. We obtained the non-local base pairing information from the DSSP database [35]. For simplicity, we only considered the non-local residue pairs in the BP1 column of the database files.

**Figure 6:**
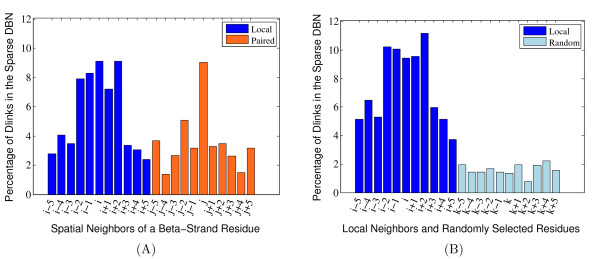
**Comparison of the local and non-local correlations in *β *strands**. (A) The figure plots the percentage of dlinks for the local and paired neighbors of a *β *strand residue at position *i*. (B) Similar to (A), except that paired neighbors are seleted randomly, rather than according to the true pattern of *β *strand pairing. The figures suggest a strong correlation between a *β *strand residue and its local neighbors as well as non-local partners (*i.e.*, those that make hydrogen bridge interaction) as compared to the remaining positions.

**Figure 7:**
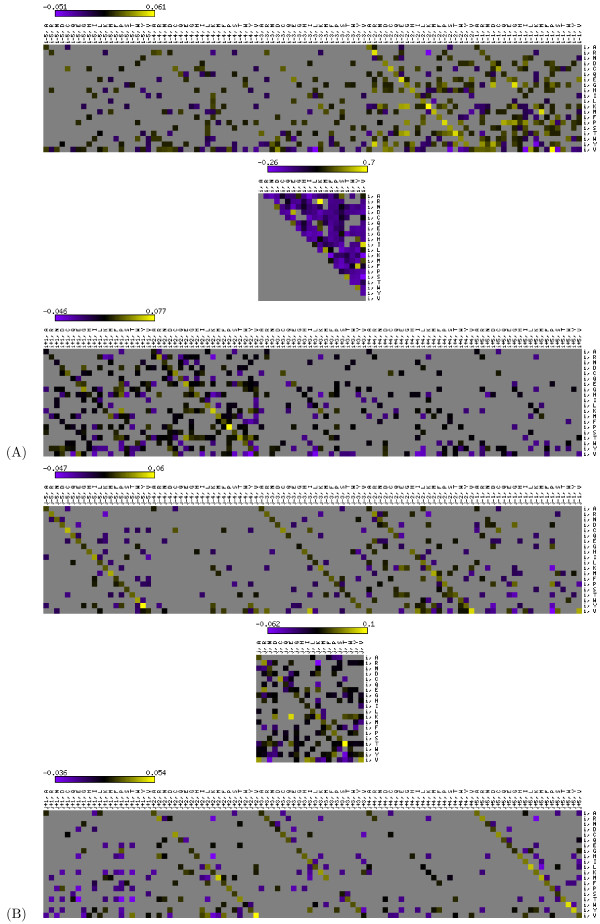
**The learned dlink values for local and non-local positions in *β *strands**. (A)The figure shows the dlink values learned from the real data for local correlations in *β *strands. The first plot illustrates the dlink values for the correlations between the *i^th ^*position and the positions at *i *- *L_AA_*, ..., *i *- 1. The second plot depicts the dlink values for the correlations within the *i^th ^*position. The third plot shows the dlink values for the correlations between the *i^th ^*position and the positions at *i *+ 1, ..., *i *+ *L_AA_*. In each plot, rows represent the 20 amino acids for the observation vector at position *i*.(B) The figure depicts the dlink values learned from the real data for non-local correlations in *β *strands such that the *β *strand residues at positions *i *and *j *are known to make a bridge interaction. The first plot illustrates the dlink values for the correlations between the *i^th ^*position and the positions at *j *- *L_AA_*, ..., *j *- 1. The second plot depicts the dlink values for the correlations between the *i^th ^*position and the *j^th ^*position. The third plot shows the dlink values for the correlations between the *i^th ^*position and the positions at *j *+ 1, ..., *j *+ *L_AA_*. In each plot, rows represent the 20 amino acids for the observation vector at position *i*. In this experiment, *L_AA _*is chosen as 5 corresponding to a window of 11 residues. In both figures, gray colored bins represent dlinks that are not present in the resulting sparse model.

After eliminating 80% of the edges from the graphical model, we observed that ~ 61% of the remaining edges are from local positions and ~ 39% are from non-local positions on the interacting *β *strand. Thus, a significant percentage of edges are retained from positions that are related to non-local interactions. The percentage of correlations that remain in the resulting model is shown in Figure 6 in a position specific manner. Figure 6(A) illustrates the degree of correlation between a *β *strand residue at position *i *and residues at flanking positions (*i *- 5 to *i *+ 5) as well as residues flanking the paired amino acid at position *j*. As a control, we repeated the experiment using randomly selected, non-local residues (positions denoted by *k*) rather than the true pairing locations. The resulting flat correlation structure is shown as bars labeled *"k" *in Figure 6(B). This control experiment shows that the correlation structure on the interacting *β *strand is much stronger than would be expected by chance. Note that the distribution we get for the local positions in Figure 6(A) is slightly different from the distribution in Figure 6(B) because in each of these experiments, we combined the set of model parameters from local positions and those that come from distal positions into a single model and sparsified this set instead of sparsifying the two sets separately. The explicit inclusion of non-local strand interactions into our model suggests that future work on improving secondary structure prediction should perform these predictions in the context of a strand interaction prediction procedure. In addition, our modified model allows us to discover significant correlations among the individual elements of the PSSMs. Figure 7(A) shows the learned edge parameters that represent local correlations, and Figure 7(B) depicts the corresponding edge parameters for the non-local correlations (between a residue and the residues surrounding its paired neighbor). This type of analysis may allow us to discover subtle relations among interacting amino acids and provide a deeper insight into protein structure. Furthermore, the edge parameters values shown in Figure 7(B) represent the propensity of possible amino acid pairs to make contacts (or interactions) and can be used as a priori information in a contact map prediction or *β *strand pairing prediction algorithm, which relies on the prediction or residue contacts of a given protein.

**Figure 8:**
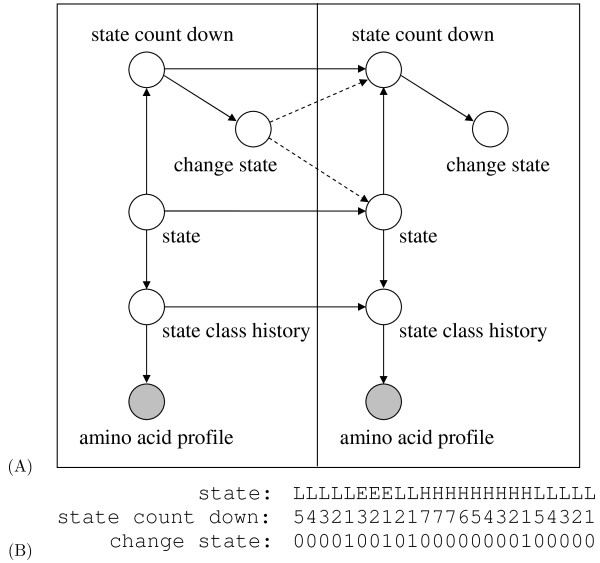
**A dynamic Bayesian network for protein secondary structure prediction**. (A) The first column shows the variables of the *prologue *(models the first amino acid) and the second column shows the variables of the *chunk *(models the second up to the last amino acid). The chunk is rolled (*i.e.*) extended to the right to get the final network. (B) An example *state *sequence and the values of *state count down *and *change state *variables for *D_max _*= 7. *state count down *and *change *state are used to control transitions from one secondary structure segment to the next and model the length distribution of segments.

## Conclusions

Our primary goal in this work was to develop and validate methods for predicting secondary structure and for training sparse DBN models. Our method outperforms the DBNN method introduced by Yao et al [5], which is a DBN cascaded by a neural network. This performance improvement results from several factors: we use PSSMs derived from HMM-profiles in addition to PSI-BLAST PSSMs, and we optimize the four hyperparameters: the amino acid profile window parameter *L_AA_*, the secondary structure label window parameter *L_SS_*, the diagonal covariance regularizer *α*, and the parameter *ω *that balances the contributions from discrete and continuous functions. Furthermore, we have demonstrated the utility of our proposed sparse model learning algorithm in three ways: (1) we can successfully eliminate 70-95% of the edge parameters in a DBN without significantly affecting the predictive accuracy of the model; (2) the learned graph structure successfully recapitulates the true underlying structure, and (3) the sparsity algorithm is able to capture local as well as non-local correlations among amino acids that are characteristic of structure formation.

The ability to reveal correlations among the elements of the observation vectors can be useful in a wide range of other problems in bioinformatics. For instance, a correlation analysis based on sparse models could be used for feature selection in other types of structure prediction algorithms such as contact map or solvent accessibility prediction. By sparsifying the feature set used by a classifier, it may be possible to jointly use additional feature representations such as PSI-BLAST and HMM-derived PSSMs to obtain even higher accuracy. Another application could be drug design simulations, where a short segment of amino acids that bind to a particular region in a target protein is designed by searching the space of possible amino acid combinations. Instead of considering all possible combinations, the procedure might be significantly simplified by concentrating on the structurally and biologically meaningful alternatives. A similar correlation analysis can also be performed to discover other types of non-local correlations, such as disulfide bonds or interactions in coiled-coil regions. Many coiled-coil type proteins are involved in important biological functions such as the regulation of gene expression and transcription factors. Moreover, the gp41 hexamer unit contains coiled-coil regions initiating the entry of HIV virus into its target cell and therefore is closely related to HIV infection [36].

Many methods exist for achieving sparse models. In comparison to methods such as ℓ_1 _regularization, our algorithm, which involves a simple truncation operation interleaved inside the standard EM algorithm [21], is quite simple. The EM algorithm is computationally efficient on the proposed model. An EM iteration for a single DBN with *L_AA _*= 9 and *L_SS _*= 6 on PDB-PC15 set of 3,824 proteins takes approximately 22 min 16 sec on a single Intel(R) Xeon(R) 2.33 GHz CPU. Furthermore, for computing predictions on test proteins, a sparse model will be much faster than the corresponding dense model, simply because the model contains fewer parameters. For example, on the SD576 benchmark, a DBN with *L_AA _*= 5 and *L_SS _*= 3 at 90% sparsity level is 3.28 times faster than the fully dense model when evaluated on a single CPU.

In future, we plan to further exploit the sparse modeling paradigm by extending our model to include additional types of observations and to identify even longer-range correlations among amino acids and secondary structure labels. As a second direction, we also plan to utilize sparse models to improve the feature set representation for other types of prediction tasks such as contact map prediction. In addition, the sparse non-local interaction patterns obtained in Figure 7(B) characterize the propensity of residue pairs to interact and can be used as features directly in a contact map prediction or *β *strand pairing prediction algorithm. Finally, the subsequent use of the secondary structure prediction method in a 3D structure prediction algorithm is also another future extension. For this purpose, it is possible to provide the posterior distribution generated from the DBNs directly as input features to a structure prediction algorithm or convert the output of the SVM to a probability [37,38].

## Methods

In the simplest variant of the secondary structure prediction problem, we are given a query protein as a series of amino acid symbols from a 20-letter alphabet. Our goal is to assign to each amino acid a structural label from a three-letter alphabet (H = helix, E = strand, L = loop). Here, we consider a variant of this problem, in which the query sequence is replaced with a position specific scoring matrix (PSSM). Transforming the amino acid sequence to a PSSM has been shown to significantly improve the predictive accuracy of secondary structure prediction [30,39,40].

### Generating position-specific scoring matrices

We use PSSMs generated by the PSI-BLAST [33] and HHMAKE [41] algorithms as input features. Each PSSM contains a smoothed statistical summary of the amino acid composition of database proteins that are closely related to the query protein. In a PSI-BLAST PSSM, the entry in row *i *and column *j *is related to the frequency of the *i^th ^*amino acid at position *j *in the alignment and is computed as(1)

Where *Q_ij _*is the estimated probability for the amino acid *i *to be at *j*, *P_i _*is the background probability (also called the background frequency) of observing amino acid *i*,*f_ij _*is the weighted frequency of amino acid *i *at position *j*, *g_i _*is the expected frequency of amino acid *i*, which is also called the "pseudocount" frequency and enables a non-zero value in the numerator when *f_ij _*is zero, *a *and *b *are scaling factors, and *λ_u _*is a constant parameter for ungapped alignments (see [42] for a more detailed description of pseudocounts).

In this work we used BLAST versions 2.2.24 and 2.2.20 and the NCBI's non-redundant (NR) database to generate PSI-BLAST PSSMs. We used BLAST version 2.2.24 and the NR database dated November, 2010, to generate the PSSM profiles for proteins in the CB513 dataset (see the "Comparison with the state-of-the-art" section). For 4 out of 513 proteins, there were no hits from the NR database and version 2.2.24 did not report any profile tables. For these proteins, we used version 2.2.20, which reports PSSMs (based on the sequence and the BLOSUM matrix) even in the absence of hits. For the SD576 dataset, we used BLAST version 2.2.24 and the NR database dated November, 2010, to generate the PSI-BLAST PSSMs used in the sections "Comparison with the state-of-the-art", "Sparsifying the model while maintaining accuracy", and "The auto-regressive section of the model contributes to accuracy". Note that the SD576 dataset contained no proteins with zero hits. In "Recovery of true sparse model structures" section, we derived the PSI-BLAST PSSMs of the SD576 dataset using BLAST version 2.2.20 and an NR database from 2009 because we performed this experiment earlier. Finally, for the PDB-PC15 dataset, we obtained the PSI-BLAST PSSMs using BLAST version 2.2.20 and the NR database dated May, 2010. The command line we used to derive the profiles from version 2.2.24 was: ./psiblast -query protein.fasta -out protein.align -out_ascii_pssm protein.pssm -num_iterations 3 -evalue 0.001 -inclusion_ethresh 1e-10 -db nr.filtered and for version 2.2.20: ./blastpgp -i protein.fasta -o protein.align -Q protein.pssm -j 3 -e 0.001 -h 1e-10 -d nr.filtered. To generate the alignments, we used the filtered NR database, which is obtained by removing the low-complexity regions, transmembrane regions, and coiled-coil segments. For this purpose, we used the "pfilt" binary of PSIPRED [43]. The PSI-BLAST and the NR database can be obtained from the help section of [44]. We derive HHMAKE PSSMs from HMM-profiles created using the HHMAKE algorithm, which is the first step of the HHsearch method [41]. Specifically, we convert the HMM-profile representation produced by HHMAKE to a PSSM by applying the following transformation:(2)

where  is the emission probability for a match state of amino acid *i *at position *j*, and  is the background probability for amino acid *i *both of which are computed by HHMAKE. Here we assume that there are *N *match states, where *N *is the number of amino acids in the query protein. This condition was satisfied for all the proteins in the benchmarks evaluated in this paper. To obtain the HMM-profiles with HHMAKE, we used the following pair of command lines: ./buildali.pl protein.fasta followed by ./hhmake protein.a3m and the HMM-profile is saved in protein.hhm. In this work, we used the HHsearch version 1.5.1 to generate profiles. The recommended database for HHMAKE is the NRE database, which is a combination of the NR and the ENV databases. The ENV database contains proteins derived from environmental sequencing projects such as "Sargasso Sea" [45] and "Mine Drainage" [46]. In addition to the type of the database used by HHMAKE, buildali.pl (the program for computing the alignments) prefers to generate the alignments first on NRE90 and then on NRE70. For this reason, we first downloaded the NR90 and ENV90 databases dated November, 2010, from [47], which are the filtered versions of NR and ENV databases at 90% and 70% identity thresholds, respectively. Then we concatenated NR90 and ENV90 to obtain NRE90. We obtained the NRE70 database similarly. The binaries used for generating the HMM-profiles can be obtained from [48].

Previous work suggests the utility of scaling the PSSM values by applying a transforming function [5,30,49]. In this work, we employ the following sigmoidal transformation to scale the PSI-BLAST and HHMAKE PSSM's:(3)

The sigmoid transforms the PSSM values into the range [0,1]. Presumably, one of the benefits of the sigmoidal transform is that it maps PSSM values in (-∞,∞) to [0,1], which normalizes the variance. Another approach is to scale the PSSM's by a linear transformation, which gives similar predictive performance as the sigmoidal transformation [5] but in this work, we did not consider this type of transformation.

### A dynamic Bayesian network for protein secondary structure prediction

We implemented the DBN shown in Figure 8(A), which is similar to the model proposed by [5]. A DBN models the generation of observation data for all possible values of hidden variables in a probabilistic framework. Thus, each node in the model represents a random variable. Our model contains five random variables. The *state *variable models the secondary structure label of an amino acid, which can be H, E, or L. This variable is observed during training and hidden when the model is employed to make predictions. The *amino acid profile *variable models the observation data, which is a 20-dimensional vector of PSSM scores (*i.e.*, a column of the PSSM) derived by running the PSI-BLAST or HHMAKE algorithms against the protein database (see the "Generating position-specific scoring matrices" section). *The state class history *variable keeps track of the current and preceding secondary structure labels. This variable is represented as a tuple with *L_SS _*+ 1 elements, where *L_SS _*+ 1 is the size of the label window, including the current label. Finally, the *state count down *and *change state *variables model the length of a secondary structure segment. When the length of a segment is less than or equal to *D_max_*, then the value of *state count down *is the number of residues from the current position to the end of the secondary structure segment. If the length of a segment is greater than *D_max _*by *k *residues, then *state count down *is set to *D_max _*for the first *k *+ 1 residues and is set to *D_max _*- 1, *D_max _*- 2, ..., 1 for the remaining residues in that segment. This allows us to estimate the length distribution of the segments that are shorter than *D_max _*+ 1 using the frequency of occurrence counts in the training set. For segments longer than *D_max _*we are fitting an exponential distribution to model the tail of the length distribution because we do not have enough data to reliably estimate this part. Details of the length distribution modeling is available in [5]. The *change state *variable simply signals when a transition to a new secondary structure segment should be made. It is set to 1 if *state count down *is 1 and 0 otherwise. An example showing the values of *state, state count down, and change state *is given in Figure 8(B). The variables *state, state count down, change state*, and *amino acid profile *are observed during training, because the true secondary structure labels are available. During testing, only the *amino acid profile *is observed, and the other variables are hidden. Therefore the DBN used for training can be slightly different from the one that is used during testing. The relations among discrete variables in the DBN are defined by conditional probability distributions (CPDs), and continuous variables are modeled by probability density functions. For instance, the state transition distribution assigns probabilities to transitions from one secondary structure state to another; distributions related to the lengths of the segments assign probability values for all possible lengths of secondary structure segments, and the observation density models the generation of the observed data.

Because of the dependencies among adjacent amino acids, the first amino acid is modeled slightly differently than the rest of the amino acids. Therefore, in Figure 8(A), the first column (*prologue*) shows the nodes for the first amino acid, and the second column (*chunk*) is a model for the rest of the amino acids. By extending the *chunk N *- 1 times to the right, we obtain the full network structure, where *N *is the number of amino acids in the protein. Detailed formulations for the CPDs that define the relations among discrete nodes can be found in [5].

In the next section, we elaborate on the probability density function that models the generation of observation data.

#### Graphical model representation

We model the PSSM observations using a linear conditional multivariate normal density,(4)

where *x_i _*is the *d*-dimensional transformed PSSM score vector for the *i^th ^*amino acid, *z_i _*is a *d L_AA_*-dimensional vector, which is a concatenation of *x*_*i*-1 _up to , *Q_i _*is the secondary structure label history defined as a tuple  with *s_i _*being the secondary structure label of the *i^th ^*amino acid, *W_q _*is a matrix of weight coefficients, *c_q _*is a shift vector, and *Σ_q _*is a conditional covariance matrix. In this formulation, the length of the PSSM profile window is *L_AA_*+1, which is the sum of *L_AA _*neighboring positions and the current (*i.e. *the *i^th^*) position. Similarly, *L_SS_*+1 is the length of the secondary structure label window. Equation 4 describes a *switching autoregressive *model--i.e., a linear dependence between an observation *x_i_*, and past observations *z_i_*, plus white noise, all dependent on a state, *q*. Note that we can convert Equation 4 into a form that allows us to represent the dependency relations among the observation vectors as a graphical model.

Following the regression approach introduced by [50] we can convert the normal density from full covariance form to diagonal covariance representation by computing a Cholesky decomposition of :(5)(6)(7)(8)

where *U_q _*is an upper triangular *d *× *d *matrix with 1's along the diagonal, Ω*_q _*is a diagonal matrix of size *d *× *d*, *V_q _*is a *d *× *d *matrix, *J_q _*is a *d *× *d *· *L_AA _*matrix, and *h_q _*is the new shift vector. In that case, our multivariate normal distribution can be re-expressed as:(9)

where , *B_q _*= [*V_q _*| *J_q_*] is of size *d *× *d *· (*L_AA _*+ 1) and is the horizontal concatenation of *V_q _*and *J_q_*, *y_i _*= [*x*_*i *_| *z_i_*] is the horizontal concatenation of *x_i _*and *z_i _*having dimension *d *· (*L_AA _*+ 1), *v_i _*is equal to *x_i _*- *B_q_z_i _*- *h_q_*, and *B_q_z_i _*+ *h_q _*is the new mean vector. Because we have a 20-dimensional Gaussian, *d *is 20.

At the end of this conversion, the linear regression relation between *x_i _*and *z_i _*can be represented as a graphical model [51], as illustrated in Figure 3. In this figure, nodes represent the individual elements of the observation vectors, *i.e.*, *x_i_*(*n*) with 1 *≤ n ≤ d*, and edges represent the dependency relations among those nodes. The figure contains 20 rows, one for each element of the observation vector, and *L_AA _*+ 1 columns representing the amino acid positions. Each edge in this graph is called a *dlink *(directed time-link) and is associated with a *dlink score b_mn_*, which is a non-zero element of the *B_q _*matrix defined in Equation 9. In this graph, the child nodes are at position *i*, which is the current position, and parent nodes are at positions *i *- *L_AA_*, ..., *i *such that the left-most column corresponds to *i - L_AA_*.

Representing the dependency relations among the elements of the observation data by a graphical model facilitates the derivation of sparse models as indeed our algorithm (below) demonstrates. Mathematically, this means that many of the elements of the final *B_q _*matrix are zero. We note that such sparse switching vector-autoregressive models have been used in the past for speech recognition problems [52], but finding a sparse structure that yielded improved performance was difficult. In practice, most such structures caused dramatic decreases in performance, and only discriminatively derived structures were beneficial. In our current case, however, where the observations are PSSMs, we see that such models can be extremely useful. We implemented the model shown in Figure 8(A) using the Graphical Models Toolkit (GMTK) [53], a C++ package for DBNs and other dynamic graphical models. GMTK represents a conditional multivariate normal density by three parameters: a mean shift vector, diagonal covariance matrix, and dlinks [53].

These correspond to the parameters in Equation 9, where  is the mean shift vector,  is the diagonal covariance matrix, and *B_q _*is the *d *× *d *· (*L_AA _*+ 1) matrix that defines dlink coefficients, which are the parameters that are sparsified by Algorithm 1.

#### Assigning a weight to the observation densities

One difference between our model and the DBN proposed by Yao *et al. *concerns the relationship between discrete and continuous variables in the model. In the "A dynamic Bayesian network for protein secondary structure prediction" section, we modeled the generation of observation data by a multivariate normal density that is conditioned on the PSSM scores at positions *i, i *- 1, ..., *i *- *L_AA _*as well as the secondary structure labels at positions *i, i *- 1, ..., *i *- *L_SS_*. Frequently, when a single DBN contains both discrete and continuous variables, the continuous densities dominate the CPDs that define the relations among the discrete variables, preventing them from contributing significantly to the overall model performance. Therefore, to reach a more balanced contribution of the CPDs and the observation density, we assign a weight to the branch that connects *state class history *and *amino acid profile *in Figure 8(A). We denote this parameter by ω and optimize it by performing an internal cross-validation procedure; i.e., within each training set in the cross-validation experiments described in "Model training, parameter optimization and testing for cross-validation" section, we first perform a secondary cross-validation to select *ω*.

#### Learning the parameters of a DBN and regularization

GMTK uses the expectation maximization (EM) algorithm [21] to learn the parameters of a DBN. Because the true secondary structure labels are available during training, EM converges to the maximum-likelihood (closed form) solution for the mean and covariance of a multivariate normal density. In this paper, we set the maximum number of EM iterations to five to learn the parameters of a DBN with fixed graphical model structures. When we learn the structure of graphical models by the sparsification algorithm described in "Learning a sparse model for a DBN" section, we perform as many EM iterations as possible until the desired level of sparsity is achieved.

During training, we applied two types of parameter regularization to better model the normal densities for rarely observed secondary structure label histories. The first regularizer adds a diagonal component to the covariance matrix, which acts as a prior on the parameters of the normal density. This regularizer was employed by [5] but is not directly implemented in the current version of GMTK. Therefore, we first converted GMTK's learned parameters to the full covariance form(10)(11)

and then carried out the following regularization:(12)

where  is the regularized covariance matrix, *α *is the regularization coefficient, and *I *is an identity matrix. We then converted the parameters back to the Cholesky form representation as formulated in Eqs. 5 to 9. We learn the *α *parameter by a cross-validation procedure. This type of regularization is known as *shrinkage *in statistical machine learning [54]. In the case of sparse models, we first apply Algorithm 1 to sparsify model parameters, add diagonal covariance regularizer as described in this section, and then eliminate any dlinks that were originally discarded by Algorithm 1 (see the "Sparsification of model parameters with a diagonal covariance component regularizer" section).

The second type of regularization stems from the fact that, when *Q_i _*is fixed, *x_i _*is a linear regression from *y_i _*with coefficients [*B_q _*| *h_q_*] as formulated in Equation 9. As with any linear regression, we can impose an ℓ_2_-regularizer on the coefficients. In GMTK, the weight of the regularizer is controlled using two parameters: λ*_d_*, which controls regularization of each element of *B_q_*; and λ*_h_*, which controls regularization of *h_q_*. ℓ_2_-norm regularization of [*B_q _*| *h_q_*] occurs during the E-step of the EM subroutine in Algorithm 1. Detailed description of the ℓ_2_-regularizer can be found in [55]. We optimized λ*_d _*and λ*_h_*, using grid search, but did not find any improvement in the overall accuracy (result not shown). In our simulations, we set λ*_h _*= 1e-4 and *λ_d _*= 500, which provided satisfactory results. Note that, in this work, we regularize both *B_q _*and *h_q_*, but sparsify only *B_q_*. We further note that ℓ_2_-regularization will not guarantee a sparse solution for *B_q_*, unlike our sparsification algorithm, introduced below.

### Learning a sparse model for a DBN

The number of edges in Figure 3 and hence the number of dlink coefficients that need to be learned during training increases linearly with respect to *L_AA _*and exponentially with respect to *L_SS_*. The latter is mainly because we have  different models for each possible value of *Q_i _*= *q*. For larger values of *L_AA _*and *L_SS_*, this exponential increase in parameters might cause problems due to the limited amount of training data. Therefore, it is useful to derive sparse graphical models, which concentrate on the strongest dependencies among the variables.

For this purpose, we developed an iterative structure learning algorithm, which is interwoven with the EM. Let *G_q _*= (*V_q_*, *E_q_*) be a graph, such as the one in Figure 3, where *V_q _*denotes the set of nodes and *E_q _*represents the set of edges in *G_q_*. Let *e_q _*be an edge in *E_q _*with a dlink score of *s*(*e_q_*), where *s*(*e_q_*) is a non-zero element of *B_q _*in Equation 9. In our case, we have  possible graphs, *i.e*, one for each normal density. For some of those Gaussians, there is no sample of the *Q_i _*variable (Equation 9) in our training data, mainly because not all possible label tuples are biologically possible. For this reason, we first scan the training set and determine the particular label tuples that have zero-occurrence counts. Then we eliminate the Gaussians that correspond to such label tuples before applying the sparsity algorithm because for those Gaussians the edge coefficients (*i.e.*, the *B_q _*matrix) will all be learned as zero. In other words, we only concentrate on sparsifying the Gaussian densities that correspond to those *Q_i _*with non-zero occurrence counts in training set. The sparsification procedure can be summarized as follows. We first do one EM iteration and learn the parameters of the multivariate normal densities. Then we sort the dlink edges in descending order with respect to their absolute values and eliminate the bottom *k*% of the edges (*i.e.*, dlinks). Note that eliminating dlinks is equivalent to setting the dlink coefficients to zero. In the next step, we initialize the next EM iteration with the new graph structure, its edge coefficients and the other learned parameters (the mean shift vector and the diagonal covariance). We iterate this procedure until the desired overall percentage of edges are eliminated. Note that we follow a greedy approach such that once we remove an edge from the graph (*i.e.*, set its edge coefficient to zero) we force that value to be zero in subsequent EM iterations. When the desired sparsity level is achieved, we learn the parameters of the final DBN by a full run of EM. At the end, we obtain a sparse DBN as well as a set of learned parameters for the multivariate normal density. A pseudocode description of this procedure is given in Algorithm 1. The sparsification of the model parameters in the presence of a diagonal covariance component regularizer is explained in the next section.

**Algorithm 1 Learning a sparse DBN**. The algorithm takes as input four parameters: the training data *D*, the percentage *k *of edges to remove at each iteration, the total fraction ℓ of edges to remove from the model, and the total number *m *of EM iterations to carry out after sparsification is complete. The final output is a trained, sparse DBN. The Initialize() subroutine initializes the  graphs such that *x_i_*(*n*) are the child nodes, and the edges are defined according to the regression relation given in the exponential term of Equation 9. The subroutine also sets the edge weights to zero, randomly initializes the mean shift vector, and sets the diagonal covariance component parameters to a fixed value. The EM subroutine carries out one iteration of the expectation maximization algorithm.

1: **procedure **LEARNSPARSEDBN(*D, k, ℓ, m*)

2:       *G *← Initialize(); *j *← 0

3:       **while ***jk *< ℓ **do**

4:          *G *← EM(*G, D*)

5:          *E *← ZeroLowest(SortByAbsVal(GetEdges(*G*)), *k*)

6:          *G *← UpdateEdges(*G, E*)

7:          *j ← j *+ 1

8:       **end while**

9:       **for ***j *← 1 ... *m ***do**

10:          *G *← EM(*G, D*)

11:       **end for**

12:       **return ***G*

13: **end procedure**

#### Sparsification of model parameters with a diagonal covariance component regularizer

After applying Algorithm 1, we convert the model parameters to the full covariance representation following Eqs. 10 and 11, add a diagonal covariance component and then convert the covariance matrices back to the Cholesky form (*i.e.*, dlink) representation following Eqs 5-8. Once we add the covariance regularizer, from each graph, we eliminate those dlinks that were originally discarded by Algorithm 1. This final elimination is necessary because when we add a diagonal covariance regularizer to the full covariance representation, dlinks that were eliminated by Algorithm 1 might have non-zero coefficients when they are converted back to the dlink representation.

### Combining multiple DBNs

Motivated by previous work [5,56], we make our predictions by combining the results from multiple DBN models. In the first model, formulated in Equation 4, we only allow dlinks from past positions. Conversely, in the second model, we reverse the PSSM profile vectors as well as the secondary structure labels and then use the same model in Figure 8(A). Effectively, the second model only allows dlinks from future positions. In both models, we use PSI-BLAST's PSSMs as the observation data. Additionally, we implement a similar pair of DBNs characterizing past and future dependencies for PSSM profiles derived using HHMAKE (see the "Generating position-specific scoring matrices" section). As a result, we have a total of four DBNs. Each model produces a marginal *a posteriori *distribution over secondary structure labels for each amino acid. The *a posteriori *probabilities can be averaged to produce a secondary structure prediction or used as features for an SVM classifier. In our simulations that analyze the predictive accuracy of sparse models (see the "Sparsifying the model while maintaining accuracy" section), we combine the DBNs by taking the average of the *a posteriori *distributions over secondary structure labels and selecting the particular label at each position that has the maximum probability. In the cross-validation experiment performed on the CB513 and SD576 benchmarks (see the "Comparison with the state-of-the-art" section), we combine the DBNs and the PSSM profiles by an SVM classifier as explained in the next section. Combining multiple models that characterize different profile representations as well as different sections of the dependency structure has a positive impact on predictive accuracy.

### Support vector machine classifier

The SVM used to combine the outputs from multiple DBNs employs a radial basis function kernel, and is trained using the LIBSVM package [57]. As the input features, we use a symmetric window of PSSM vectors derived from PSI-BLAST and HHMAKE, as well as a window of marginal *a posteriori *probabilities that are generated from the DBNs described in the "Combining multiple DBNs" section. For simplicity, we set the lengths of the PSSM and the posterior probability windows to be five, which is similar in size to the *L_AA _*window parameter optimized for DBNs (see the "Model training, parameter optimization and testing for cross-validation" section). Our feature set contains the following *a posteriori *distributions: (1) average of posterior probabilities from the four DBNs, (2) average of posterior probabilities from past dependency and future dependency DBNs that use PSI-BLAST PSSMs, (3) average of posterior probabilities from past dependency and future dependency DBNs that use HHMAKE PSSMs. This gives a total of 539 features.

For positions at which the feature window exceeds the boundaries of a protein (*i.e.*, those that are close to the N- or C-terminus), we include zeros to the feature set. In our SVM classifier, we performed 5-fold internal cross-validation on a randomly downsampled version of each training set to optimize the cost parameter *C *and the radial basis function kernel width parameter γ. In this procedure, the downsampling rate is set to 5 and a grid search is performed such that *C *∈ {2^-5^, 2^-3^, ..., 2^5^} and *γ *∈ {2^-7^, 2^-13^, ..., 2^2^}. Then we use the selected parameters to train an SVM classifier on the training set and generate predictions on the test set. LIBSVM uses a one-against-one method to generate predictions for more than two classes.

### Model training, parameter optimization and testing for cross-validation

For each train/test split of the cross-validation experiment in the "Comparison with the state-of-the-art" section, we randomly divided each training set into two such that the first half is used to optimize and train the DBNs and the second half the SVM. Each DBN requires the specification of four hyperparameters: the dependency parameters *L_AA _*and *L_SS_*, the diagonal covariance regularizer *α*, and the discrete/continuous weighting parameter *ω*. In our cross-validation experiment with the CB513 benchmark, we performed internal cross-validation on each training set allocated for DBNs and selected the hyperparameters of the DBNs that yielded the highest amino acid level accuracy. To avoid an expensive search over the full, four-dimensional search space, we performed the optimization in a step-wise fashion. First, we optimized the *L_AA _*and *L_SS _*parameters, fixing *α *= 0.01 and *ω *= 1.0. In this step, we considered a grid of values for *L_AA _*and *L_SS_*, *i.e.*, *L_AA _*from 0 to 10 and *L_SS _*from 0 to 6. Second, we fixed *L_AA _*and *L_SS _*to their optimum values and searched for the best *α *parameter. Third, we fixed *α *to its optimum value and optimized the *ω*. For *α *and *ω*, we did a binary search, where we started with values from {0.1, 0.2, ..., 1.0}, selected the optimum on this grid and then selected a finer grid of values, with increments of 0.01, around that optimum. After selecting the optimum on this finer grid, we selected a third grid of values with increments of 0.001 around that optimum and searched for the best values of the hyperparameters. Once we obtained the optimum values for the hyperparameters we repeated this optimization procedure starting with the second round of optimization for *L_AA _*and *L_SS _*fixing *α *and *ω *to their optimum values from the first round, followed by the reoptimization of *α *and *ω*. For DBNs that use PSSMs derived from PSI-BLAST, the hyperparameter optimization for the CB513 set yielded values in the vicinity of *L_AA _*= 4, *L_SS _*= 2 both for the past and future DBN models. The mean and standard deviation of the *α *parameter was (0.0424, 0.014) for the past DBN and (0.0450, 0.012) for the future DBN. For the *ω *parameter, these statistics were (0.567, 0.094) for the past DBN and (0.525, 0.081) for the future DBN. Similarly, for DBNs that use HHMAKE PSSMs, the optimum values were approximately *L_AA _*= 3, *L_SS _*= 2 for the past DBN and *L_AA _*= 4, *L_SS _*= 2 for the future DBN. The mean and standard deviation of the *α *parameter was (0.020, 0.087) for the past DBN and (0.012, 0.035) for the future DBN. For the *ω *parameter, the mean and standard deviation values were (0.420, 0.032) for the past DBN and (0.367, 0.031) for the future DBN. We observed that, the optimized values are more consistent across different cross-validation splits for DBNs that use PSI-BLAST based PSSMs as compared to DBNs that use HHMAKE PSSMs. We hypothesize that this difference arises because PSI-BLAST PSSMs are regularized using pseudocounts, whereas HHMAKE PSSMs are not. Including pseudocounts in the HHMAKE PSSMs might make the estimated hyperparameters more consistent across different cross-validation splits because a pseudocount will smooth the estimated PSSM values by assigning a background measure to the cases with zero-occurrence *i.e.*, cases with no hits to a particular amino acid in a column of the multiple alignment block; however, we did not explicitly test this hypothesis. Detailed description of pseudocounts can be found in [42] and in [58]. Similar values for the hyperparameters are obtained for the SD576 set (data not shown). In our DBN model, *L_AA_*, *L_SS _*and *α *control the model complexity, determining whether the classifier will underfit or overfit to a given training set. For instance, as we increase *L_AA _*or *L_SS_*, after a certain point, we will start observing a decrease in the predictive accuracy, which is known as over-fitting (a detailed analysis of the performance with respect to to *L_AA _*and *L_SS _*can be found in Yao et al [5]). On the other hand, the covariance component regularizer *α *allows us to smooth the model (high values enable more smoothing) and reduce over-fitting. Therefore, the accuracy with respect to these hyperparameters will be close to a concave function. In our simulations, we also observed a similar concave behavior for the *ω *parameter (data not shown). To optimize *C *and γ, which are the two hyperparameters of the SVM, we performed an internal cross-validation on the training set allocated for the SVM (see the "Support vector machine classifier" section). The optimum values were around *C *= 1.0 and γ = 0.00781 for the CB513 and SD576 benchmarks.

After optimizing the hyperparameters, for each train/test split, we trained the DBNs using the first half of the training set and generated marginal *a posteriori *probability distributions for proteins in the second half of the training set as well as proteins on the test set. Then we trained the SVM on the second half of the training set and predicted the secondary structure of proteins in the test set. Pseudocode for the nested cross-validation procedure is given in Algorithm 2. Detailed descriptions of the hyperparameter optimization algorithms are also available in Supplementary Algorithms 1, 2, and 3 (see the Additional file 1).

**Algorithm 2 Pseudocode for cross-validation with hyperparameter optimization by internal cross-validation**. The algorithm takes as input four parameters: the dataset *D*, the number of cross-validation folds *K*, the number of folds for internal cross-validation *K_int_*, and the set of hyperparameters Θ = (*L_AA_, L_SS_, α, ω*). For each train/test split, the algorithm optimizes the hyperparameters of the DBNs by doing internal cross-validation on the training set. Then it trains the DBNs on the training set with the optimized set of parameters and computes predictions on the test set.

1: **procedure **NESTEDCROSSVALIDATION(*D, K, K_int_*, Θ)

2:    Split dataset *D *into *K *(*train, test*) sets

3:    Split each *train *set into two (*train_DBN _, train_SV M _*)

4:    **for **each (*train_DBN _, train_SV M _, test*) **do**

5:       Split *train_DBN _*into *K_int _*(*subtrain, subtest*) sets

6:       **for **each hyperparameter *θ *∈ Θ **do**

7:          **for **each (*subtrain, subtest*) **do**

8:             Train DBNs on *subtrain *and predict on *subtest*

9:          **end for**

10:          Compute the accuracy on *train_DBN _*for *θ*

11:       **end for**

12:       Select θ* with the best accuracy for a given *train_DBN_*

13:       Train DBNs on *train_DBN _*with θ* and predict on *train_SV M _*and *test*

14:       Train SVM on *train_SV M _*and predict on *test*

15:    **end for**

16:    Return the accuracy on *D*

17: **end procedure**

### PDB-PC15 dataset

To obtain the PDB-PC15 dataset, we used the following set of criteria in PISCES server [34]: percent identity threshold of 15%, resolution cutoff of 2.5 Å, and R-value cutoff of 1.0. We also used PISCES to filter out non-X-ray and C*α*-only structures and to remove short (< 40 amino acids) and long (> 10000 amino acids) chains. In the resulting set of proteins, we replaced chemically modified residues (*i.e.*, the ones annotated as "X" by the DSSP algorithm [23]) with the unmodified versions taken from the PDB. Finally, we assigned a secondary structure label to each amino acid from the DSSP database [35]. We mapped the 8-state representation of secondary structure labels to 3-states by applying the following conversion rule: H, G, I to H; E, B to E and S, T, ' ' to L. The final version of our dataset contains 3,824 chains and 792,146 amino acids.

For the *β *strand analysis, we extracted the *β *strand segments only, eliminating the particular segments that contain amino acids with zero bridge interactions and those that are of length one (*β*-bridge residues). The final set contained 16,927 *β *strand segments with a minimum segment length of 2, maximum of 18 and average of 4.

### Model for analyzing correlations in *β *strands

To allow the analysis of correlations among *β *strand residues, we update the definition of the multivariate normal density in Equation 4 as(13)

where *x_i _*is the observation vector of a *β *strand residue *r_i_*, and *x_j _*is the observation vector of the residue *r_j_*, which makes a bridge interaction with *r_i_*. This formulation allows us to fit a multivariate normal density to *p*(*x_i _*| *z_i_*, *Q_i _*= *q*) as in Equation 4 and analyze the correlation relations between *r_i _*and its spatial neighbors.

## Authors' contributions

WSN and JB coordinated the study. ZA implemented the algorithms and evaluated their performance. JB introduced the sparsity algorithm for DBNs and the representation of the PSSMs by graphical models. ZA introduced the secondary structure prediction method and the HHMAKE PSSMs. ZA and WSN wrote and edited the manuscript. AS and JB provided support on technical aspects and edited the manuscript. All authors read and approved the final manuscript.

## Supplementary Material

Additional file 1 **Cross-validation and hyperparameter optimization**. Detailed descriptions of the algorithms for cross-validation and hyperparameter optimization by internal cross-validation. Click here for file
