# Supplementary material for: Learning sparse models for a dynamic Bayesian network classifier of protein secondary structure
Source: BMC Bioinformatics. 2011 May 13;12:154. doi: 10.1186/1471-2105-12-154 (PMC3118164; doi:10.1186/1471-2105-12-154)
Supplement: Additional file 1 — Cross-validation and hyperparameter optimization. Detailed descriptions of the algorithms for cross-validation and hyperparameter optimization by internal cross-validation. [file 1471-2105-12-154-S1.PDF]

Supplement to "Learning sparse models for a dynamic Bayesian network classifier of protein secondary structure"

Zafer Aydin*[1], Ajit Singh[2] , Jeff Bilmes[2] and William S Noble*[1,3]

[1]Department of Genome Sciences, University of Washington, Seattle, WA 98195, USA
[2]Department of Electrical Engineering, University of Washington, Seattle, WA 98195, USA
[3]Department of Computer Science and Engineering, University of Washington, Seattle, WA 98195, USA

Email: Zafer Aydin*- zafer@u.washington.edu; Ajit Singh - ajit@ee.washington.edu; Jeff Bilmes - bilmes@ee.washington.edu; William S Noble*- william-noble@u.washington.edu;

*Corresponding author

Supplementary Algorithm 1 Cross validation with hyperparameter optimization. The algorithm takes as input three parameters: D is the dataset on which the cross-validation experiment is performed, K is the number of iterations for a K-fold cross-validation, and K_{int} is the number of iterations for internal cross-validations. The `RandomlySplit(.)` subroutine randomly splits the input dataset D into K training sets (T and U) and K test sets (V) such that T_i is the ith labeled set of proteins that is used to train DBNs, U_i is the ith labeled set of proteins that is used to train the SVM, and V_i is the ith labeled set of proteins that is used to predict the secondary structure where i is the index for the cross-validation iteration. The number of amino acids contained in a dataset is represented by the cardinality symbol. For example, the number of amino acids in D is denoted by $|D|$. M_1 specifies the type of the PSSM that is used in DBNs such that $M_1 = p$ denotes PSI-BLAST PSSMs, $M_1 = h$ represents HHMAKE PSSMs, and $M_1 = A_1$ refers to the average model that is obtained by combining posterior distributions from $M_1 = p$ and $M_2 = h$. M_2 determines the direction of the dependency window for the PSSM profiles in DBNs such that if $M_2 = N$ then a given position depends on past positions, *i.e.*, those that are closer to the N-terminal of the protein, if $M_2 = C$ then a given position depends on future positions, *i.e.*, those that are closer to the C-terminal of the protein, and $M_2 = A_2$ refers to the average model that is obtained by combining posterior distributions from $M_2 = N$ and $M_2 = C$. $L_{AA}^{(M_1,M_2)}$, $L_{SS}^{(M_1,M_2)}$, $\alpha^{(M_1,M_2)}$, $\omega^{(M_1,M_2)}$ are the hyper-parameters of the DBN for a given (M_1, M_2) pair. Because (M_1, M_2) can take four possible values, we have a total of four DBNs. `OptimizeDBN(.)` subroutine performs internal cross-validation experiments on the training set allocated for DBNs and optimizes the hyper-parameters as explained in Supplementary Algorithms 2 and 3. `InitializeDBN(.)` allocates $3^{L_{SS}+1}$ multivariate normal densities (Gaussians) for a DBN, initializes the graphical model structure of each Gaussian, and sets the weight parameter ω. B represents a DBN model. `GetPSSM(.)` returns the set of PSSMs for a given dataset and PSSM type. `EM(.)` learns the parameters of the DBN by the EM algorithm. $\theta_B^{(M_1,M_2)}$ represents the parameters of the DBN such as state transition distributions, distributions that specifiy length contraints, and mean, covariance parameters of the multivariate normal densities. `GetCovariance(.)` returns a covariance matrix from a given set of DBN's parameters and the index of the Gaussian. `UpdateParameters(.)` updates the set of DBN's parameters with the regularized covariance matrix. `JT(.)` executes the "junction-tree" algorithm and generates the marginal *a posteriori* probability distribution of secondary structure labels denoted as $P(u_j^i|Y_i, \theta_B^{(M_1,M_2)})$ for each amino acid such that j is the amino acid index in Y_i, which is the ith set of PSSMs used to train the SVM and u_j^i is the secondary structure label variable for that amino acid. Similarly, $P(v_k^i|Z_i, \theta_B^{(M_1,M_2)})$ is the marginal *a posteriori* probability distribution for proteins in Z_i, which is the set of PSSMs that is used as the ith test set. `TrainSVM(.)` learns the parameters of the SVM classifier and `PredSVM(.)` predicts secondary structure labels by the SVM. s is the variable that represents the secondary structure labels of all the amino acids in D. `EvaluateAccuracy(.)` computes the accuracy measures by comparing the predicted secondary structure labels to the true labels in D. Λ is the set of accuracy measures.

procedure CrossValidation(D, K, K_{int})
  $(T, U, V) \leftarrow$ RandomlySplit(D);
  for $i \leftarrow 1 \ldots K$ **do**
    for $M_1 \leftarrow \{p, h\}$ **do**
      for $M_2 \leftarrow \{N, C\}$ **do**
        $(L_{AA}^{(M_1,M_2)}, L_{SS}^{(M_1,M_2)}, \alpha^{(M_1,M_2)}, \omega^{(M_1,M_2)}) \leftarrow$ OptimizeDBN(T_i, K_{int}, M_1, M_2)
        $B \leftarrow$ InitializeDBN($L_{AA}^{(M_1,M_2)}, L_{SS}^{(M_1,M_2)}, \omega^{(M_1,M_2)}, M_2$)
        $X_i \leftarrow$ GetPSSM(T_i, M_1)
        $\theta_B^{(M_1,M_2)} \leftarrow$ EM(X_i, B)
        for $m \leftarrow 1 \ldots 3^{L_{SS}+1}$ **do**
          $\Sigma_m \leftarrow$ GetCovariance($\theta_B^{(M_1,M_2)}, m$)
          $\Sigma_m^{reg} \leftarrow (1-\alpha)\Sigma_m + \alpha^{(M_1,M_2)} I$
          $\theta_B^{(M_1,M_2)} \leftarrow$ UpdateParameters($\theta_B^{(M_1,M_2)}, \Sigma_m^{reg}, m$)
        end for
▷ The algorithm continues on the next page...

$Y_i \leftarrow \text{GetPSSM}(U_i, M_1)$
$Z_i \leftarrow \text{GetPSSM}(V_i, M_1)$
$P(u_j^i | Y_i, \theta_B^{(M_1,M_2)}) \leftarrow \text{JT}(\theta_B^{(M_1,M_2)}, Y_i), \quad 1 \le j \le |U_i|$
$P(v_k^i | Z_i, \theta_B^{(M_1,M_2)}) \leftarrow \text{JT}(\theta_B^{(M_1,M_2)}, Z_i), \quad 1 \le k \le |V_i|$
end for
end for
for $j \leftarrow 1 \ldots |U_i|$ **do**
$P(u_j^i | Y_i, \theta_B^{(p,A_2)}) \leftarrow \Big(P(u_j^i | Y_i, \theta_B^{(p,N)}) + P(u_j^i | Y_i, \theta_B^{(p,C)}) \Big) /2$
$P(u_j^i | Y_i, \theta_B^{(h,A_2)}) \leftarrow \Big(P(u_j^i | Y_i, \theta_B^{(h,N)}) + P(u_j^i | Y_i, \theta_B^{(h,C)}) \Big) /2$
$P(u_j^i | Y_i, \theta_B^{(A_1,A_2)}) \leftarrow \Big(P(u_j^i | Y_i, \theta_B^{(p,A_2)}) + P(u_j^i | Y_i, \theta_B^{(h,A_2)}) \Big) /2$
end for
for $j \leftarrow 1 \ldots |V_i|$ **do**
$P(v_j^i | Z_i, \theta_B^{(p,A_2)}) \leftarrow \Big(P(v_j^i | Z_i, \theta_B^{(p,N)}) + P(v_j^i | Z_i, \theta_B^{(p,C)}) \Big) /2$
$P(v_j^i | Z_i, \theta_B^{(h,A_2)}) \leftarrow \Big(P(v_j^i | Z_i, \theta_B^{(h,N)}) + P(v_j^i | Z_i, \theta_B^{(h,C)}) \Big) /2$
$P(v_j^i | Z_i, \theta_B^{(A_1,A_2)}) \leftarrow \Big(P(v_j^i | Z_i, \theta_B^{(p,A_2)}) + P(v_j^i | Z_i, \theta_B^{(h,A_2)}) \Big) /2$
end for
$\theta_S \leftarrow \text{TrainSVM}(Y_i, P(u_j^i | Y_i, \theta_B^{(p,A_2)}), P(u_j^i | Y_i, \theta_B^{(h,A_2)}), P(u_j^i | Y_i, \theta_B^{(A_1,A_2)})), \quad 1 \le j \le |U_i|$
$s_j \leftarrow \text{PredSVM}(\theta_S, Z_i, P(v_j^i | Z_i, \theta_B^{(p,A_2)}), P(v_j^i | Z_i, \theta_B^{(h,A_2)}), P(v_j^i | Z_i, \theta_B^{(A_1,A_2)})), \quad 1 \le j \le |V_i|$
end for
$s \leftarrow \cup_k s_k$
$\Lambda \leftarrow \text{EvaluateAccuracy}(s, D)$
end procedure

Supplementary Algorithm 2 Hyperparameter optimization for a DBN by internal cross-validation. The algorithm takes as input four parameters: T is the dataset on which the cross-validation for parameter optimization is going to be performed, K_{int} is the number of cross-validation iterations, M_1, and M_2 specify the types of the DBN model as explained in Supplementary Algorithm 1. The algorithm returns the optimized hyperparameters of the DBN: $L^*_{AA}, L^*_{SS}, \alpha^*, \omega^*$. Q_3 is the overall accuracy measure on T. `RandomlySplit2(.)` subroutine randomly splits the input dataset T into K_{int} training sets denoted by E and K_{int} test sets R such that E_i is the ith labeled set of proteins that is used to train DBNs and R_i is the ith labeled set of proteins that is used to predict the secondary structure where i is the index for the cross-validation iteration. η represents the parameters of the DBN including the discrete probability distributions and the continuous density functions. Similarly, γ is the updated set of DBN's parameters such that the covariance matrices in γ are regularized by a diagonal covariance component. `OptimizeWeight(.)` optimizes the weight parameter α or ω as described in Supplementary Algorithm 3. `EvaluateQ3(.)` computes the Q_3 measure on a set of predicted labels and a given set of true labels. k is the index for the optimization steps, which are performed twice. Other parameters and subroutines are explained in Supplementary Algorithm 1.

---

procedure OptimizeDBN(T, K_{int}, M_1, M_2)
  $(E, R) \leftarrow$ RandomlySplit2(T);
  $\alpha^* \leftarrow 0.0$
  $\omega^* \leftarrow 1.0$
  $Q_3^* \leftarrow 0$
  for $k \leftarrow 1, 2$ **do**
    for $L_{AA} \leftarrow 0 \ldots 10$ **do**
      for $L_{SS} \leftarrow 0 \ldots 6$ **do**
        $B \leftarrow$ InitializeDBN($L_{AA}, L_{SS}, \omega^*, M_2$)
        for $i \leftarrow 1 \ldots K_{int}$ **do**
          $X_i \leftarrow$ GetPSSM(E_i, M_1)
          $Y_i \leftarrow$ GetPSSM(R_i, M_1)
          $\eta \leftarrow$ EM(X_i, B)
          for $m \leftarrow 1 \ldots 3^{L_{SS}+1}$ **do**
            $\Sigma_m \leftarrow$ GetCovariance(η, m)
            $\Sigma_m^{reg} \leftarrow (1-\alpha^*)\Sigma_m + \alpha^* I$
            $\gamma \leftarrow$ UpdateParameters(η, Σ_m^{reg}, m)
          end for
          $P(s_j|Y_i,\gamma) \leftarrow$ JT(γ, Y_i, B), $\quad 1 \le j \le |R_i|$
          $s_j^* \leftarrow \arg\max_{s_j} P(s_j|Y_i,\gamma)$
        end for
        $s^* \leftarrow \cup_j s_j^*$
        $Q_3 \leftarrow$ EvaluateQ3(s^*, T)
        if $Q_3 > Q_3^*$ **then**
          $Q_3^* \leftarrow Q_3$
          $L^*_{AA} \leftarrow L_{AA}$
          $L^*_{SS} \leftarrow L_{SS}$
        end if
      end for
    end for
    $\alpha^* \leftarrow$ OptimizeWeight($T, K_{int}, L^*_{AA}, L^*_{SS}, \omega^*, M_2, 1$)
    $\omega^* \leftarrow$ OptimizeWeight($T, K_{int}, L^*_{AA}, L^*_{SS}, \alpha^*, M_2, 2$)
  end for
end procedure

Supplementary Algorithm 3 Weight optimization for a DBN. The algorithm takes as input eight parameters: T is the dataset on which the cross-validation for parameter optimization is going to be performed, K_{int} is the number of cross-validation iterations, L_{AA} is the PSSM profile window parameter, L_{SS} is the secondary structure tuple parameter, δ is the parameter that is used to initialize α or ω, M_1, and M_2 specify the types of the DBN model as explained in Supplementary Algorithm 1, and $flag$ specifies whether the α or the ω parameter is going to be optimized. λ is the variable that keeps track of the parameter that is optimized. The algorithm returns the optimized hyperparameter of interest of the DBN (α^* or ω^*). $\triangle$ is the step increment that is used to select the value of α or ω. η represents the parameters of the DBN including the discrete probability distributions and the continuous density functions. Similarly, γ is the updated set of DBN's parameters such that the covariance matrices in γ are regularized by a diagonal covariance component. Other parameters and subroutines are explained in Supplementary Algorithms 1 and 2.

procedure OptimizeWeight($T, K_{int}, L_{AA}, L_{SS}, \delta, M_1, M_2, flag$)
    $(E, R) \leftarrow$ RandomlySplit2(T);
    $\lambda_c \leftarrow 0$
    $\lambda^* \leftarrow 0$
    $\triangle \leftarrow 0.1$
    $Q_3^* \leftarrow 0$
    for $j \leftarrow 1, 2, 3$ **do**
        for $n \leftarrow -9 \dots 9$ **do**
            $\lambda \leftarrow \lambda_c + n \times \triangle$
            if $\lambda < 0$ **then**
                continue
            end if
            if $flag = 1$ **then**
                $\omega \leftarrow \delta$
                $\alpha \leftarrow \lambda$
            else if $flag = 2$ **then**
                $\omega \leftarrow \lambda$
                $\alpha \leftarrow \delta$
            end if
            $B \leftarrow$ InitializeDBN($L_{AA}, L_{SS}, \omega, M_2$)
            for $i \leftarrow 1 \dots K_{int}$ **do**
                $X_i \leftarrow$ GetPSSM(E_i, M_1)
                $Y_i \leftarrow$ GetPSSM(R_i, M_1)
                $\eta \leftarrow$ EM(X_i, B)
                for $m \leftarrow 1 \dots 3^{L_{SS}+1}$ **do**
                    $\Sigma_m \leftarrow$ GetCovariance(η, m)
                    $\Sigma_m^{reg} \leftarrow (1-\alpha)\Sigma_m + \alpha I$
                    $\gamma \leftarrow$ UpdateParameters(η, Σ_m^{reg}, m)
                end for
                $P(s_j|Y_i, \gamma) \leftarrow \text{JT}(\gamma, Y_i, B), \quad 1 \le j \le |R_i|$
                $s_j^* \leftarrow \arg\max_{s_j} P(s_j|Y_i, \gamma)$
            end for
            $s^* \leftarrow \cup_j s_j^*$
            $Q_3 \leftarrow$ EvaluateQ3(s^*, T)

▷ The algorithm continues on the next page...

if $Q_3 > Q_3^*$ **then**
$Q_3^* \leftarrow Q_3$
$\lambda^* \leftarrow \lambda$
end if
end for
$\triangle \leftarrow \triangle / 10.0$
$\lambda_c \leftarrow \lambda^*$
end for
end procedure
